# Upregulation of mucin glycoprotein MUC1 in the progression to esophageal adenocarcinoma and therapeutic potential with a targeted photoactive antibody-drug conjugate

**DOI:** 10.18632/oncotarget.15340

**Published:** 2017-02-15

**Authors:** Mohammed Adil Butt, Hayley Pye, Rehan J. Haidry, Dahmane Oukrif, Saif-U-Rehman Khan, Ignazio Puccio, Michael Gandy, Halla W. Reinert, Ellie Bloom, Mohammed Rashid, Gokhan Yahioglu, Mahendra P. Deonarain, Rifat Hamoudi, Manuel Rodriguez-Justo, Marco R. Novelli, Laurence B. Lovat

**Affiliations:** ^1^ Department for Tissue & Energy, University College London, London, UK; ^2^ Upper Gastrointestinal Service, University College London Hospitals NHS Foundation Trust, London, UK; ^3^ Department of Pathology, University College London, London, UK; ^4^ Department of Oncology, UCL Cancer Institute, London, UK; ^5^ Antikor BioPharma, Stevenage Bioscience Catalyst, Hertfordshire, UK; ^6^ Department of Chemistry, Imperial College London, London, UK

**Keywords:** antibody-drug conjugate, mucins, Barrett’s esophagus, esophageal adenocarcinoma, photodynamic therapy

## Abstract

**Background:**

Mucin glycoprotein 1 (MUC1) is a glycosylated transmembrane protein on epithelial cells. We investigate MUC1 as a therapeutic target in Barrett’s epithelium (BE) and esophageal adenocarcinoma (EA) and provide proof of concept for a light based therapy targeting MUC1.

**RESULTS:**

MUC1 was present in 21% and 30% of significantly enriched pathways comparing BE and EA to squamous epithelium respectively. MUC1 gene expression was x2.3 and x2.2 higher in BE (p=<0.001) and EA (p=0.03). MUC1 immunohistochemical expression increased during progression to EA and followed tumor invasion. HuHMFG1 based photosensitive antibody drug conjugates (ADC) showed cell internalization, MUC1 selective and light-dependent cytotoxicity (p=0.0006) and superior toxicity over photosensitizer alone (p=0.0022).

**Methods:**

Gene set enrichment analysis (GSEA) evaluated pathways during BE and EA development and quantified MUC1 gene expression. Immunohistochemistry and flow cytometry evaluated the anti-MUC1 antibody HuHMFG1 in esophageal cells of varying pathological grade. Confocal microscopy examined HuHMFG1 internalization and HuHMFG1 ADCs were created to deliver a MUC1 targeted phototoxic payload.

**Conclusions:**

MUC1 is a promising target in EA. Molecular and light based targeting of MUC1 with a photosensitive ADC is effective *in vitro* and after development may enable treatment of locoregional tumors endoscopically.

## INTRODUCTION

Despite progress in the treatment of other cancers, the 5-year survival of esophageal adenocarcinoma (EA) remains low at around 15% [1]. Barrett’s epithelium is a premalignant change that increases the risk of developing EA 30-100 fold above that for the general population [2, 3]. Eradication of Barrett’s epithelium significantly reduces the risk of developing EA [4]. Identifying new therapeutic targets for Barrett’s epithelium and EA is of vital clinical importance. Within the field of esophagogastric adenocarcinoma HER2 is the only therapeutic biomarker to be incorporated into widespread clinical practice. The HER2-targeting antibody Trastuzumab when used in combination with chemotherapy has been shown to improve progression-free and overall survival in HER2 positive gastric and gastroesophageal junction cancer patients [5]. HER2 overexpression occurs in approximately 13-23% of esophagogastric cancers but expression can be heterogeneous [6, 7]. An ideal therapeutic target would be stable and present in a higher proportion of tumors.

The mucin MUC1 is a densely glycosylated transmembrane protein anchored to the apical surface of many epithelia including the breast, ovary, pancreas, airway and gastrointestinal tract. The extracellular subunit of MUC1 has a ‘variable number tandem repeat’ (VNTR) region which consists of a repeating 20 amino acid sequence mediating heavy O-linked glycosylation [8]. MUC1 has an important extracellular role in cell surface lubrication and the clearance of debris and pathogens. Its intracellular signaling is linked to the ErbB, fibroblast growth factor receptor 3 and p53 pathways, which are implicated in cancer development [9]. MUC1 is overexpressed in a diverse range of carcinomas. In progression to cancer, MUC1 protein expression generally increases, alters location and is coupled with aberrant glycosylation [8, 10]. Previous studies of MUC1 expression in the premalignant changes of EA are inconsistent [11–13]. Up-regulation of MUC1 has been linked to bile acids exposure in gastroesophageal reflux, a condition connected to the development of Barrett’s epithelium and EA [14]. Others have associated a single nucleotide polymorphism in the MUC1 gene to a reduced risk of other upper gastrointestinal cancers [15].

HuHMFG1 is an antibody against MUC1 that has been tested in clinical trials for breast cancer [16–18]. Targeting using HuHMFG1 was ineffective alone but the antibody was well tolerated and had a good safety profile [19]. Later studies used the radiolabeled anti-MUC1 antibody (yttrium-90-AS1402) in ovarian cancer after de-bulking surgery. Administration led to endogenous production of anti-MUC1 IgG in some patients, but there was no survival benefit in those in whom this occurred [20]. HuHMFG1 undergoes cell internalization [21] and was considered as a vehicle for an antibody-drug conjugate (ADC) approach using the potent cytotoxin, calicheamicin. Reasonable efficacy was seen but in this example the overall therapeutic window was low as calicheamicin was not well tolerated at the higher doses [22]. ADCs are a well-established and clinically-successful approach to cancer therapy but target and payload selection are key in developing drugs with high efficacy and tolerability, i.e. a high therapeutic index (TI). With the right payload, an anti MUC1 ADC could have great potential.

Photodynamic therapy (PDT) is an ideal modality for application to ADC, particularly where there is some degree of normal tissue expression and hence the TI is low. PDT is already an established treatment modality for dysplastic Barrett’s epithelium [23]. It has also shown utility in the treatment of cancers of the prostate, lung, pancreas, bile duct, oral cavity and skin [24, 25]. PDT involves the administration of a photosensitizer (PS) and its activation locally using light to cause cellular destruction via intracellular free radicals and/or reactive oxygen species [26–29]. As there is little effect on connective tissue, it preserves luminal integrity when used in the digestive tract [29]. Though not inherent to PDT itself, the first generation photosensitizers approved for clinical use such as porfimer sodium suffered from suboptimal pharmacokinetic/biodistribution profile and poor tumor selectivity. This led to low potency and off target photosensitivity, resulting in severe sunburn and the scarring of internal organs in some patients [23]. These limitations can be inherent to any therapeutic molecule but by chemical modification and/or combination with other molecules it is now possible to avoid them [30–34]. Photoimmunotherapy is one such approach using a niche ADC where PS are targeted with antibodies (photoimmunoconjugates) [33, 35–38].

This study aims to highlight the role MUC1 plays in the progression to EA. It further examines how MUC1 expression and glycosylation are altered during esophageal malignant transformation and later locoregional invasion. Finally proof of principle data for the mechanism and *in vitro* efficacy of a MUC1 targeting ADC using PDT is shown.

## RESULTS

### Identification of MUC1 as a biomarker in the development of EA

**Figure 1:**
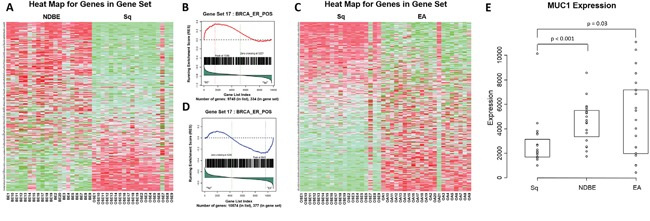
Gene set enrichment and microarray analysis of MUC1 in the progression to esophageal adenocarcinoma Heat map **A**. and an example probability plot **B**. of the gene set enrichment analysis (GSEA) for non-dysplastic Barrett’s esophagus (NDBE) vs normal squamous esophageal epithelium (Sq). Heat map **C**. and an example probability plot **D**. of the GSEA for esophageal adenocarcinoma (EA) vs Sq. GSEA detail in supplementary (Supplementary Figure 1) and evaluated with Kolmogorov-Smirnoff test. Microarray analysis **E**.; raw expression values of MUC1 mRNA in Sq, NDBE and EA tissues, results show a 2.3 fold increase in MUC1 expression at the mRNA level in NDBE compared to Sq (Mann-Whitney; p < 0.001) and 2.2 fold increase in EA compared to Sq (Mann-Whitney; p = 0.03). Box plot presented as median and interquartile range.

MUC1 was linked to the progression to EA using gene set enrichment analysis (GSEA). Within the GSEA two groups of upper GI samples were compared; the comparison of non-dysplastic Barrett’s esophagus (NDBE) to normal esophageal squamous epithelium (Sq) gave 47 pathways that were enriched in NDBE compared to Sq, of which 28 were significant and of these 21% included MUC1. Comparison of EA to Sq gave 49 pathways enriched in EA compared to Sq of which 27 pathways were significant and of these 30% included MUC1 (Figure 1 and Supplementary Figure 1). This recurrent appearance of MUC1 in the significant pathways suggests involvement in the transition of normal esophageal tissue to malignancy. Some of the most significant pathways included both MUC1 and HER2. To see if the MUC1 gene was up regulated during cancer progression the data was mined using the Affymetrix probe for MUC1 to retrieve raw gene expression values. When compared to Sq, mRNA levels in NDBE show a 2.3 fold increase in MUC1 expression (p < 0.001), while mRNA levels in EA showed an increase in both the range of expression as well as an overall 2.2 fold increase in MUC1 expression (p = 0.03) (Figure 1).

### MUC1 glycoprotein tissue staining

**Figure 2:**
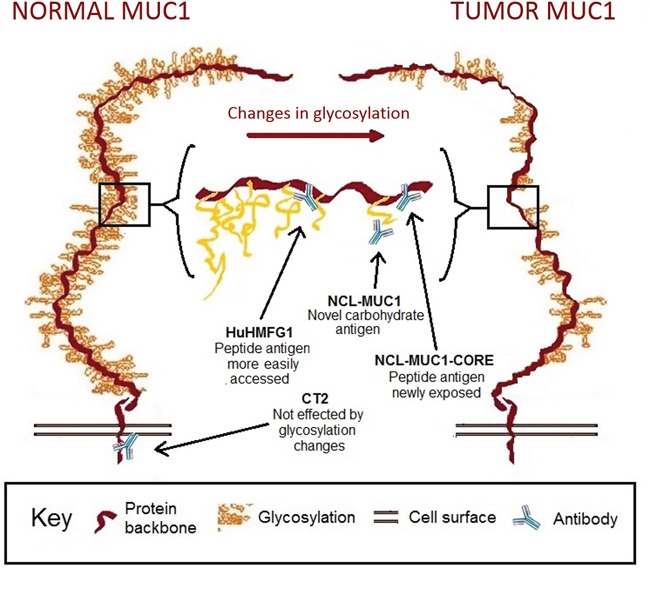
Representation of MUC1 receptor structure in normal and tumor epithelium with binding sites for selected antibodies Representation of MUC1 receptor glycosylation in normal and tumor epithelium. NCL-MUC1 binds a sialic acid on the glycosylated side chain, while NCL-MUC-1-CORE and HuHMFG1 bind the extracellular peptide backbone. The extracellular target antigens can be hidden in fully glycosylated normal tissue, but become increasingly exposed in cancer due to aberrant glycosylation. CT2 targets the intracellular cytoplasmic tail of MUC1.

**Figure 3:**
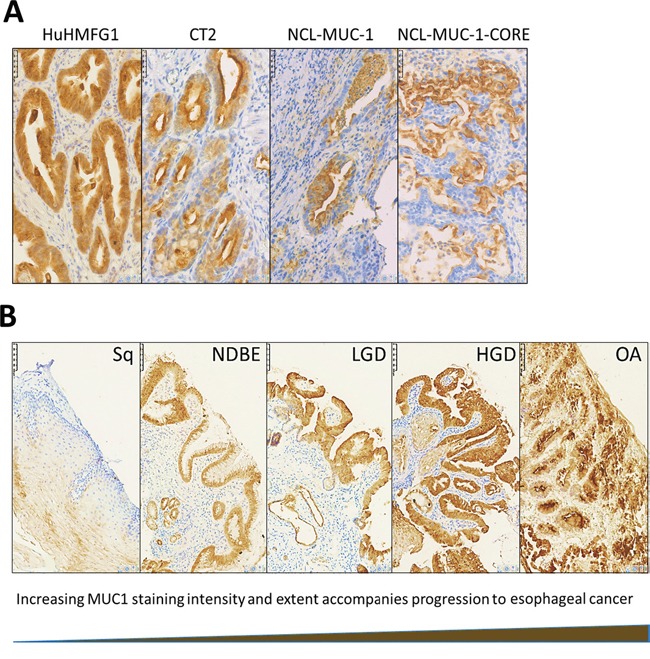
Immunohistochemical staining patterns with anti-MUC1 antibodies in high grade dysplasia and HuHMFG1 staining in the squamous-metaplasia-dysplasia-carcinoma sequence **A**. Immunohistochemical images of high-grade dysplasia in Barrett’s epithelium stained with four anti-MUC1 antibodies (brown), and hematoxylin (blue). **B**. HuHMFG1 staining in normal esophageal squamous epithelium (Sq), non-dysplastic Barrett’s esophagus (NDBE), low-grade dysplasia (LGD), high grade dysplasia (HGD) and invasive esophageal adenocarcinoma (EA). An increase in the intensity of staining is seen as pathological grades progress. Staining also follows the direction of epithelial maturation from basement membrane toward the lumen. In higher pathological grades, staining is seen throughout the epithelial layer.

Four antibodies against different epitopes of MUC1 (Figure 2) were used to stain patient samples representing various stages toward progression to cancer; Sq epithelium, NDBE, low-grade dysplasia (LGD), high grade dysplasia (HGD) and invasive esophageal adenocarcinoma (EA). HuHMFG1 immunostaining was mostly membranous and cytoplasmic with additional nuclear staining in highly expressing samples. CT2 and NCL-MUC-1 stained predominantly the apical membrane with mild cytoplasmic positivity. NCL-MUC-1-CORE staining was focused on the luminal surface of cells. In all cases binding was limited to the epithelial cell layer. The intensity of HuHMFG1 staining increased in the progression to EA, and towards the more differentiated superficial epithelial cells (Figure 3).

**Figure 4:**
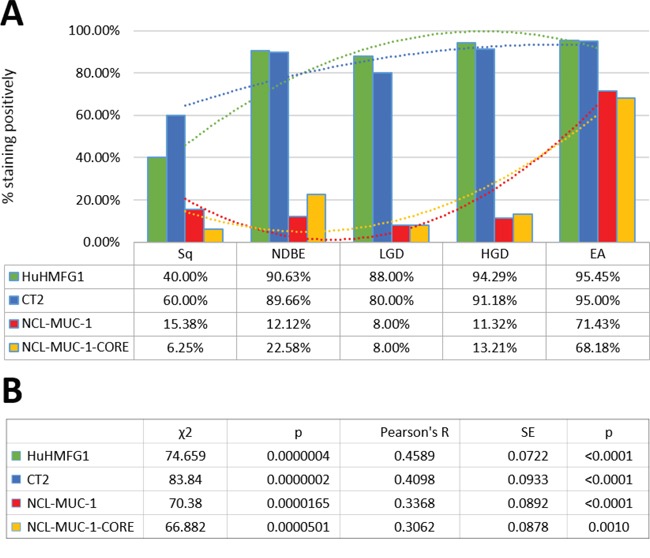
Levels of expression of four MUC1 epitopes in the squamous-metaplasia-dysplasia-carcinoma sequence **A**. The anti-MUC1 antibodies HuHMFG1, CT2, NCL-MUC-1 and NCL-MUC-1-CORE were evaluated by immunohistochemistry in esophageal tissue from incremental pathological grades including normal squamous epithelium (Sq), non-dysplastic Barrett’s esophagus (NDBE), low-grade dysplasia (LGD), high grade dysplasia (HGD) and invasive esophageal adenocarcinoma (EA). Positivity was defined by 2+/3+ intensity staining in ≥10% of the pathology examined. The proportion of positive samples for each tissue is shown with respectively colored polynomial lines of best fit. HuHMFG1 and CT2 staining increase at the metaplastic (NBDE) stage, whereas NCL antibodies increase staining after development of EA. **B**. All antibodies showed significant expression differences during progression to cancer (χ2 test; p < 0.00005), with all having a significant trend of increasing positivity (Pearson’s R; p < 0.001).

HuHMFG1 and CT2 binding increased from moderate in squamous mucosa to high levels in NDBE. NCL-MUC-1 and NCL-MUC-1-CORE maintained low binding levels in normal and dysplastic tissue but increased to high levels of binding in EA tissue (Figure 4). An alternative representation (Supplementary Figure 2) shows the Allred score of individual samples and is presented to show the heterogeneity of all four antibodies across all pathological grades. Due to the recognized EA risk of dysplastic columnar epithelium, and a desire to target it therapeutically [4, 39], HuHMFG1 was selected as the optimum antibody to take forward for therapeutic development over CT2 as the latter is not as suitable for ADC development due to its intracellular location. Since it was shown HuHMFG1 bound some normal epithelium, development as a photoimmunoconjugate was chosen as the use of light to selectively activate the drug in a local area could be used to avoid the majority of normal epithelium which can be distinguished endoscopically.

HuHMFG1 staining at the optimized concentration was sensitive (95% of cancers were identified as positive) but not specific (40% of normal tissue also stained positive). Specificity for HGD and EA could be demonstrated by using a less antibody (0% staining in Sq, NDBE and LGD) however this was at the expense of substantially reduced sensitivity (positive staining in HGD and EA fell to ∼20%) (Supplementary Figure 3).

**Figure 5:**
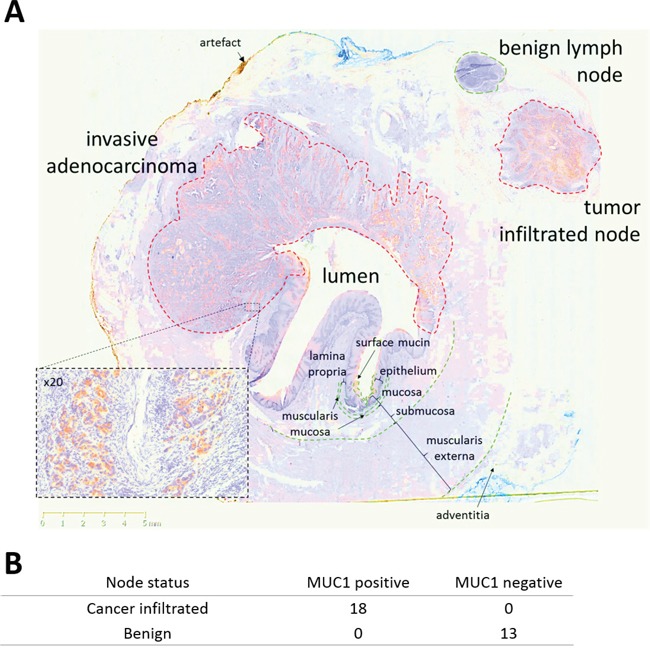
HuHMFG1 staining MUC1 in invasive esophageal adenocarcinoma and locoregional lymph node metastases **A**. A transverse esophagectomy section from a patient with invasive esophageal adenocarcinoma (EA) stained with HuHMFG1 (brown) and hematoxylin (blue). HuHMFG1 stains surface mucin, invasive EA as it invades into muscularis externa and only the local tumor infiltrated lymph node. Normal mucosa and a benign local lymph node did not stain positively. A x20 magnification from a representative tumor region is inset. **B**. Analysis of 31 locoregional nodes resected from 11 patients highlight positive expression of MUC1 by HuHMFG1 in all malignant but no benign lymph nodes (Fishers exact; p <0.0001).

Staining of resection specimens demonstrated that HuHMFG1 does not bind normal connective tissue, vascular or muscular structures of the esophagus. The epithelial specificity of HuHMFG1 is demonstrated exquisitely in Figure 5 in which a whole esophagus transverse section taken from a patient with EA was stained with HuHMFG1. This section includes 2 lymph nodes, one infiltrated with cancer and one free of disease. HuHMFG1 selectively stains only the infiltrated node. To confirm this pattern in lymph nodes, staining was extended to a panel of 11 EA resection specimens with 31 associated locoregional nodes. HuHMFG1 staining was positive in all 18 tumor infiltrated lymph nodes and negative in all 13 benign nodes (Fishers exact p <0.0001). This reinforces the choice for HuHMFG1 for therapeutically application as any off target effects in normal areas would spare connective, muscular and vascular tissue and damaged normal squamous mucosa can regrow [40].

### HuHMFG1 potential as a therapeutic antibody in living cells

**Figure 6:**
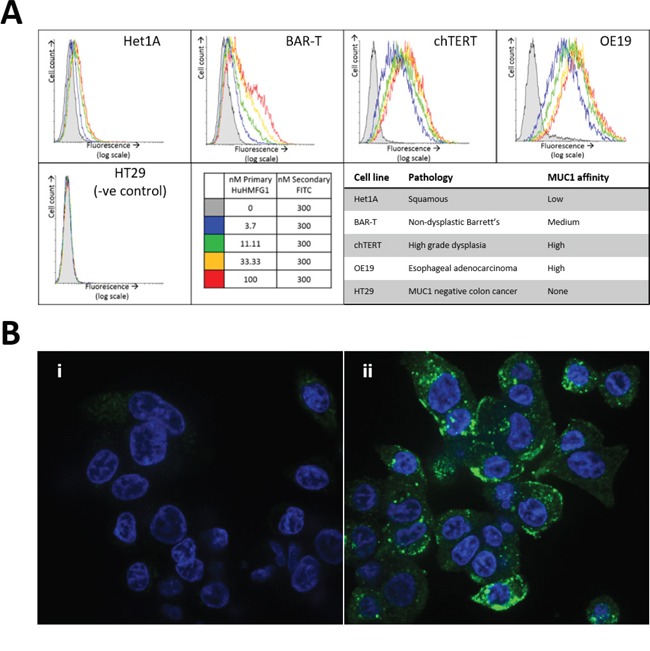
MUC-1 expression in esophageal cell lines of various pathological grades and internalization into esophageal adenocarcinoma **A**. Binding of the antibody HuHMFG1 *in vitro* to esophageal cell lines originally isolated from tissue of various pathological grades was carried out using flow cytometry. Cells were incubated with varying concentrations of HuHMFG1 antibody which was detected using a FITC conjugated anti-human IgG secondary fluorescent antibody (shown in green) shown alongside nuclei staining with DAPI (shown in blue). An increase in fluorescence represents more HuHMFG1 bound to each cell and the saturation of the fluorescent signal indicates cell surface receptor saturation. HuHMFG1 did not bind the colonic line HT29 (negative control). It bound at a low level in normal squamous and non-dysplastic Barrett’s esophagus (NDBE) and a high level in high grade dysplasia (HGD) and esophageal adenocarcinoma (EA). **B**. Confocal microscopy images showing OE19 cells exposed to HuHMFG1 (i) or media alone (ii). OE19 internalize HuHMFG1 and the intracellular localization pattern is punctate.

To confirm binding to native antigen in living cells, HuHMFG1 was tested *in vitro* on a panel of esophageal cell lines derived from each stage in the squamous-metaplasia-dysplasia-carcinoma sequence. All EA lines showed a dose dependent increase in cell binding of HuHMFG1 to a point of cell surface receptor saturation; HuHMFG1 binding was at a low level in normal squamous cells (Het1A) but then incrementally increased through NDBE cells (BAR-T), HGD cells (chTERT) with the highest level of binding seen in EA cells (OE19) (Figure 6).

For an effective ADC, it is preferable but not required that it underwent intracellular internalization after binding. Using confocal microscopy, internalization of HuHMFG1 into an EA cell line (OE19) was shown (Figure 6). The punctate intracellular pattern is similar to the pattern seen in previous work where endosomal co-localization of HuHMFG1 was demonstrated in a breast cancer cell line [21].

**Figure 7:**
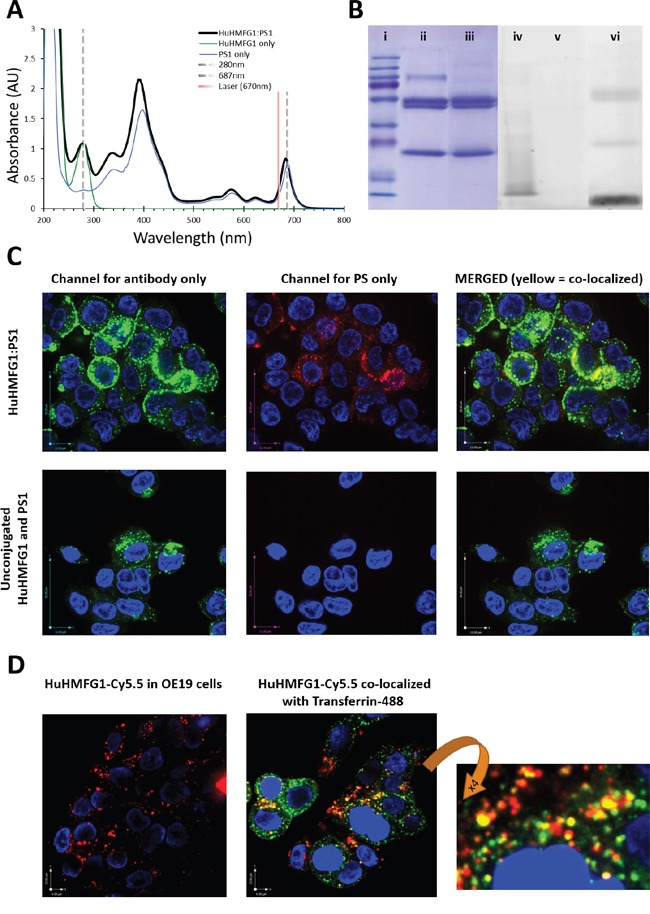
Photophysical characterization and internalization of photoactive MUC1 targeting antibody drug conjugates **A**. Absorbance spectra of HuHMFG1:PS1 antibody drug conjugate (ADC) highlights peak absorbance at 683nm (red spectral region) for laser excitation. Shown are any photophysical shifts away from the absorbent spectra of free antibody or free PS1 (spectra normalized to 280nm or 687nm respectively). **B**. SDS page gel showing proportion of covalently coupled antibody to free photosensitizer (PS1) in the ADC mixture; *(i-iii)* Coomassie stained protein gel; *(i)* molecular weight markers, *(ii)* HuHMFG1 and *(iii)* HuHMFG1:PS1 ADC. *(iv-vi)* Image of the same SDS gel before Coomassie staining for the PS1 dye via UV fluorescence; *(iv)* molecular weight markers, *(v)* HuHMFG1 and *(vi)* HuHMFG1:PS1 ADC. Covalently bound photosensitizer PS1 is that seen at the same height as antibody protein. **C**. Confocal microscopy images showing OE19 cells exposed to HuHMFG1 and PS1 in either a conjugated form or free un-conjugated form showing the covalently bound photosensitizer and antibody components remain co-localized after internalization. HuHMFG1 (in green) nuclei staining (in blue) PS1 (in red). **D**. HuHMFG1 was conjugated to a non-toxic dye Cy5.5 and OE19 cells exposed to the HuHMFG1:Cy5.5 conjugate with and without a marker of recycling endosomal localization. Cells were co-stained with the nuclear stain DAPI shown in blue. Cy5.5 fluorescence is shown in red, endosomal marker is shown in green and the partial co-localization of HuHMFG1 and endosomal marker is shown in yellow.

To prove the targetable therapeutic potential of HuHMFG1, an ADC of the antibody was made via NHS ester mediated amide conjugation of photosensitive drug molecules (PS1) to available lysine amino acids [41, 42]. UV-VIS spectrometry of the final HuHMFG1:PS1 ADC dissolved in PBS confirmed peak absorption at 683nm for laser activation and approximately 7 PS1 photosensitizers were coupled on average onto each HuHMFG1 antibody (Figure 7). Digital image analysis of reducing SDS-PAGE separation of the conjugate indicated 52% of the PS1 was conjugated to HuHMFG1 via a covalent amide bond, and 48% was conjugated via non-covalent interactions. The non-covalent material in the conjugate was tightly bound and not dissociated in biological buffers or with the addition of low level detergent during the purification process. Upon conjugation small shifts in the absorbance spectra of PS1 were observed; the two main PS1 peaks at 398nm and 687nm shifted 4-5nm into the blue, and the ratio between the two main peaks changed from 2.2 in the free PS1 to 2.6 in the conjugated form. There were no shifts in absorbance observed between the free and conjugated antibody at 280nm (Figure 7).

Confocal microscopy was used to confirm HuHMFG1:PS1 ADC internalization into OE19 cells. Both the drug (PS1) and antibody (HuHMFG1) parts of the conjugate remain co-localized after internalization in a similar pattern to that seen with unconjugated HuHMFG1 (Figure 7). Due to the photoactive nature of the drug, carrying out live cell imaging induces cell death. In an attempt to co-localize the ADC with a marker of endosomal localization, a nontoxic ADC was produced using the same reaction conditions but in which non-toxic Cy5.5 dye molecules were covalently conjugated to HuHMFG1 instead of PS1. Spectral analysis of the new HuHMFG1:Cy5.5 ADC show the antibody had an average of 5 dye molecules covalently bound per antibody and when run on SDS PAGE, the conjugate showed 30% non-covalently bound Cy5.5 (data not shown). Results showed partial co-localization of the HuHMFG1:Cy5.5 conjugate with transferrin a marker of the recycling endosomal pathway. This could suggest HuHMFG1 conjugates are internalized during endocytosis but then delivered to another compartment, i.e. the lysosome compartment (Figure 7).

**Figure 8:**
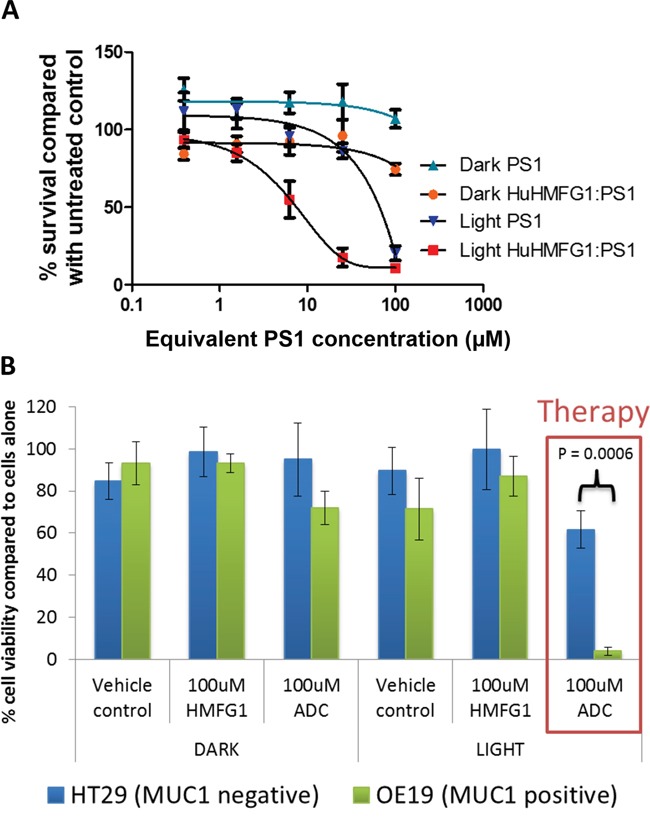
Light dependent cytotoxicity and superior efficacy over equivalent free photosensitizer of a MUC1 specific photoactive antibody drug conjugate **A**. The cytotoxicity of HuHMFG1:PS1 ADC, was compared with equivalent free photosensitizer (PS1) concentrations in light and dark conditions in OE19 cells. Light activation was by laser at 0.33J/cm2 at 670nm over 10 seconds. Light activated ADC cytotoxicity was significantly more effective than light activated PS1 cytotoxicity (linear regression of dose response curves with F test for comparison; p=0.0002; F=26.09). **B**. The cytotoxic efficacy of HuHMFG1:PS1 ADC, HuHMFG1 antibody alone and vehicle control were compared in a MUC1 positive line (OE19) and a MUC1 negative line (HT29) with and without light activation. Light activation involved an energy exposure of 0.33J/cm2 at 670nm over 10 seconds. Light activated ADC cytotoxicity was significantly more effective in the MUC1 positive line compared to the MUC1 negative control (Students t-test; p=0.0006). No significant cytotoxic effect was seen in either line with the vehicle control, antibody alone or ADC without light activation.

The phototoxic ADC HuHMFG1:PS1 was investigated for its light dependent cytotoxicity with an *in vitro* MUC1 positive EA cell line. OE19 cells were incubated in the dark with a range of doses of either HuHMFG1:PS1 or the equivalent concentration of free PS1 molecules. Cells were then washed and either left in the dark or irradiated with low intensity laser light at 670nm [0.33J/cm^2^ over 10seconds] (Figure 8A). Despite the non-covalently bound material, light-dependent cytotoxicity of the HuHMFG1:PS1 ADC was seen to be significantly more potent than equivalent amounts of PS1 alone (linear regression p=0.0022, F=26.09). No significant cytotoxicity was seen with either drug in the dark (linear regression p=0.7335, F=0.1273) or between the vehicle control (2% DMSO) and cells in media only (p=0.12) (data not shown). The top HuHMFG1:PS1 dose was then used to compare cytotoxicity in MUC1 positive OE19 cells to a MUC1 negative cell line HT29 (Figure 8B). The phototoxic efficacy of the HuHMFG1:PS1 ADC was significantly greater in MUC1 positive OE19 cells than negative HT29 cells (t test p<0.0006). No cytotoxicity was seen in either line with the unconjugated antibody or with the ADC in the absence of light.

## DISCUSSION

GSEA looks at cellular pathways focusing on groups of genes that share common biological function, chromosomal location, or regulatory pathways. GSEA found MUC1 was identified as a protein involved with, and up-regulated in, the progression from normal squamous epithelium to invasive EA. To confirm the GSEA results, and to clarify some of the variations in MUC1 IHC profiles previously reported in the literature, 4 different antibodies recognizing different epitopes of MUC1 were tested in a panel of human biopsy samples.

The NCL-MUC-1-CORE antibody binds directly to the VNTR protein backbone where normal mucin glycosylation levels sterically hinder antibody binding. Sagara and colleagues had similar findings with a different MUC1 backbone binding antibody (DF3). They too showed it did not bind normal squamous esophageal epithelium [43]. Strong binding of this antibody in EA is most likely due to a tumor associated MUC1 reduction in glycan chain length and density similar to that seen in breast cancer [44]. NCL-MUC-1 showed a similar binding pattern to NCL-MUC-1-CORE.NCL-MUC-1 binds a sialylated amino acid attached to a carbohydrate linked to the peptide backbone and like NCL-MUC-1-CORE this epitope was shown to be either exposed or upregulated much later in the progression pathway. Other mucin associated Sialyl-Tn antigens have similarly been associated with the final stages of malignant transformation in squamous cell esophageal carcinoma [45]. Quantification of these antibodies reflects staining intensity and the amount of antibody bound. With MUC1 this staining intensity will not correlate with MUC1 expression at the molecular level, this is due to variation in the number of tandem repeats between individuals [46]. Though variation does exist, for the purpose of clinical translation we expect MUC1 will follow the example set by Trastuzumab in HER2 positive esophagogastric cancer and use a similar IHC staining intensity cut-off to identify which individuals would respond well to antibody treatment [5].

HuHMFG1 and CT2 demonstrate persistently high levels of binding in all pathological grades from NDBE to EA making them suitable for ADC development for early and locally advanced esophageal neoplasia. CT2 is an intracellular epitope so further development was focused on HuHMFG1. Though HuHMFG1 bound a proportion of normal epithelium, its specificity to the epithelial layer is important therapeutically. Damaged mucosa can re-grow but serious damage occurs when therapy reaches deeper muscle layers and can cause esophageal strictures [23, 47]. Re-epithelialization with neosquamous epithelium is likely to follow HuHMFG1 treatment strategies in pre-invasive disease, similar to regeneration seen following ablative esophageal therapies [4]. HuHMFG1 also offers potential to treat a selection of established invasive cancers, including those with locoregional lymph node spread, particularly if a photosensitizing drug is used which is activated by deep red light. At 670nm, light penetrates more deeply into tissue than at 633nm, used to activate first generation PS such as porfimer sodium. Under optimal conditions, depth of necrosis can also extend up to five times the light penetration [48]. In bladder tumors, 673nm light penetrates 5.03mm to create PDT effect up to 25mm from the urothelial surface [49]. When administered into the lumen of the esophagus, a similar depth of PDT effect is envisaged allowing for treatment of locally infiltrated lymph nodes.

HuHMFG1 was shown to bind a panel of living esophageal cells throughout the pathological grades in a similar pattern to that observed with IHC. To provide proof-of-concept of HuHMFG1 as an ADC a conjugate with a photoactive-drug was produced. Photodynamic therapy (PDT) is particularly relevant for EA as laser light is easily applied via endoscopy. Targeting photosensitizers with HuHMFG1 would improve PDT selectivity. PS1 (previously developed as compound 1) is a second generation photosensitizer based on the chlorophyll derivative PPa. It has been chemically manipulated to include a bioconjugation handle and a series of short PEG-like chains to increase its water solubility [41]. PS1 was conjugated to HuHMFG1 via NHS mediated amide bond formation to exposed lysine amino acid residues. The ADC produced was shown to contain an average of 7 PS1 molecules per antibody but around half of these were non-covalently bound. Since the method for estimating conjugation ratio uses extinction coefficients based on the absorbance spectra of the free PS1 dye, the small shifts in absorbance upon conjugation may compromise the validity of the resulting ratio. Further work would have to be done to address this before the ADC is taken further. The additional non-covalently bound material is a problem seen elsewhere with PDT-ADCs and would hinder further development as it stands. It would likely cause batch-to-batch inconsistencies and make for poor chemical and biological reproducibility alongside a loss of selectivity of the final product [50]. Despite some of the PS1 being non-covalently bound, the photosensitizer and antibody seem to remain co-localized *in vitro*. The intracellular localization of the photoimmunoconjugate is critical to the mechanism of action and would rather be established more fully and where possible done with the final drug, not a non-toxic equivalent as we have shown, if this was to be taken further. For future translation we plan to improve purity by using smaller antibody fragments with optimized lysine spacing and an alternative conjugation strategy with more hydrophilic PS. This would have advantages from a pharmacokinetic and manufacturing point of view [33]. We have successfully applied this technique using a HER2 targeting antibody Fab fragment [51]. We believe despite these issues with purity, the cytotoxicity results presented offer a promising proof of concept for a MUC targeted ADC. A low dose of laser light, only 0.33J/cm^2^, was used to make it translatable to the lower level of light that can be clinically delivered deeper into tissue. The wavelength of light used was 670nm and so use of a laser closer matched to the peak absorbance of PS1 (Figure 7) would hopefully improve cytotoxicity. HuHMFG1:PS1 demonstrated light dependent cytotoxicity that was significantly more effective for both the MUC1 positive cell line than for the MUC1 negative cell line and more potent than equivalent free PS1 photosensitizer.

This paper confirms how MUC1 is upregulated during progression with gene set enrichment analysis, immunohistochemistry and flow cytometry in esophageal tissues and cell lines taken from discrete histological grades. It clarifies previously conflicting data with regards to the detection of epitopes differentially expressed on MUC1 due to glycosylation changes. It shows how MUC1 expression is maintained by glandular cells throughout the metaplasia-dysplasia-carcinoma sequence, binding only the epithelial layer in early disease. In advanced disease, expression is maintained by glandular cells as they invade the submucosa, muscular layer and into metastases. HuHMFG1 was shown to positively bind a high proportion of cases from non-dysplastic Barrett’s epithelium, through degrees of dysplasia to invasive EA. This suggests HuHMFG1 may have excellent therapeutic potential. To confirm this, we showed HuHMFG1 binding in live cells and developed first in class proof-of-concept MUC1 targeting photoactive ADCs. We postulated on its therapeutic mechanism and confirmed its cytotoxic efficacy for the future translation of this antibody into the clinical arena.

## MATERIALS AND METHODS

### Gene set enrichment analysis

Microarray data was obtained from Gene Expression Omnibus data from Wang et al [52]. The data set included 19 normal esophageal squamous epithelium, 20 NDBE and 21 EA samples. Gene set enrichment analysis was carried out as previously described on around 4000 cellular pathways using the Kolmogorov-Smirnoff test [53 ]. Recurrent genes in the most important pathways in transition from squamous to NDBE and squamous to EA were identified. The MUC1 gene probe 213693_s_at was then used to mine additional gene expression microarray data [52, 54–56] for raw mRNA expression levels using Affymetrix, and compared with Mann-Whitney test.

### Tissue panel

A panel of formalin fixed paraffin embedded (FFPE) esophageal specimens of varying pathological grades was identified from the University College London Hospital upper gastrointestinal clinical database. The pathology sample chosen was of the highest grade the patient had at the time of sampling. Ethical approval was obtained from the UK Research Ethics Committee (EC13.13; 08/H808/8; 08/H0714/27). Esophageal tissue samples were selected from 123 patients containing, in order of disease severity; normal squamous tissue (n=15), NDBE (n=29), low grade dysplasia (n=25;), high grade dysplasia (n=34) and invasive EA (n=20). Sections from selected samples were stained with Hematoxylin and Eosin (H&E) to confirm the reported pathological grade by two expert GI pathologists (MN, MRJ). H&E staining was performed using standard protocols.

### Immunohistochemistry

In order to examine MUC1 glycoprotein expression during progression to EA, four antibodies against distinct areas and/or glycoforms were tested. The mouse monoclonal antibody NCL-MUC1 binds a sialylated amino acid attached to a carbohydrate linked to the PDTRPAP region of the VNTR [57]. The humanized monoclonal antibody HuHMFG1 binds a glycan linked PDTR amino acid sequence on the VNTR [58]. The mouse monoclonal antibody NCL-MUC-1-CORE binds directly to the TRPAPG amino acid sequence on the VNTR [57]. The hamster monoclonal antibody CT2 targets the intracellular SSLSYTNPAVAATSANL amino acid sequence on the cytoplasmic tail of MUC1 [59] Binding of antibodies to the antigens near or on the VNTR can be sterically hindered in fully glycosylated normal MUC1, but become increasingly exposed in cancer due to aberrant glycosylation [60] [61] (Figure 1).

Immunohistochemical (IHC) techniques were optimized to maintain strongly positive tissue staining in the absence of background staining with antibodies at the following primary concentrations. NCL-MUC-1 (1:500; Leica-Novocastra), HuHMFG1 (1:1000 [10μg/mL]; Antisoma, UK) NCL-MUC-1-CORE (1:500; Leica-Novocastra) and CT2 (1:500; gifted by Professor Sandra Gendler, Mayo Clinic, USA). Staining was carried out using heat-induced epitope retrieval in pH 6.0 sodium citrate buffer (Sigma-Aldrich, S4641). Endogenous peroxidase (Leica, RE7157) and non-specific protein activity (Leica, RE7158) were blocked prior incubation with primary antibody. The humanized antibody HuHMFG1 required initial biotinylation (ThermoFisher, 21335) followed by incubation with ExtrAvidin Peroxidase (Sigma-Aldrich E2886). All other slides were incubated with post-primary block (Leica RE7159) and polymer (Leica RE7161). Slides were then developed with 3,3-diaminobenzadine tetrahydrochloride as a chromogen (Leica RE7162), counterstained with hematoxylin, dehydrated in graded ethanol and mounted in distyrene plasticizer xylene (Sigma Aldrich 06522).

All slides were scored by two expert GI pathologists (MN, MRJ). Positive MUC1 cases were defined as those staining 2+/3+ intensity in ≥10% of the pathology examined, following the established classification adopted for HER2 [5]. The Allred system was also used to characterize staining in more detail. Intensity was scored as negative (0), mild (1), moderate (2+) and strong (3+), and proportion of tissues positively stained as negative (0), <1% (1), 1-<10% (2), 10-<33% (3), 33-<66% (4) and ≥66% (5) [62]. Statistical tests including chi squared, Pearson’s R and Linear regression analysis were performed using IBM^®^ SPSS^®^ statistics Version 22 (IBM Corporation).

### Cell culture

Het1A obtained from ATCC (October 2014) is a human cell line established from an area of normal esophageal epithelium that has been SV40 large T antigen-immortalised [63, 64]. BAR-T gifted by Prof Rhonda Souza (UT Southwestern, USA) is a human cell line established from an area of non-neoplastic Barrett’s that has been telomerase-immortalised [65]. ChTERT (CP-52731) gifted by Dr Stuart McDonald (Barts Cancer Institute, UK) is a human epithelial cell line established from an area of high grade dysplastic Barrett’s esophagus that has also been telomerase-immortalised [66]. OE19, obtained from the ECACC (May 2014) is a human epithelial cell line established from a stage three moderately differentiated esophageal adenocarcinoma at the esophageal gastric junction [67]. These cells were compared to human Caucasian colon adenocarcinoma cell line (HT-29) gifted by Prof Marilena Louzidou (Royal Free Hospital, UK) as a negative control, as it does not bind HuHMFG1. All lines were cultured either according to ECACC/ATCC guidelines or their original publication. All cells were confirmed mycoplasma free.

### Flow cytometry

Cell lines were detached with Accutase (Millipore SCR005) or a 0.05%Trypsin/0.02% EDTA/0.5%PVP solution for Het1A cells. Approx. 200,000 cells per sample were washed and incubated in 50ul on ice with varying concentrations of HuHMFG1. After 1 hour cells were again washed and exposed to 300nM α-Human IgG (FAB specific) FITC conjugate (Sigma-Aldrich F5512) on ice for 30 minutes before two final washes. All steps carried out in FC buffer; PBS + 2% FCS + 1mM EDTA. Flow cytometry was carried out on a Beckman-Coulter Cyan ADP, Cells underwent laser excitation at 488nm and emission was recorded between 510nm and 550nm.

### Confocal microscopy

OE19 cells were plated in a Lab-Tek 8 well chambered borosilicate cover glass (NUNC 155411) at 10,000 cells/well and cultured at 37°C/5%CO2. Figure 6= Cells were cultured for 4hrs in media with and without 0.5μM HuHMFG1. Figure 7C= Cells were cultured for 2.5hrs in media with either 0.5μM HuHMFG1:PS1 in the ADC conjugated form or 0.5μM unconjugated HuHMFG1 plus the equivalent amount of free PS1 that was present in the ADC. *Figure 7D*= Cells were cultured for 5hrs with 0.3μM HuHMFG1:Cy5.5 with or without additional 0.3uM Transferrin Alexa Fluor 488 (Molecular Probes, Life Technologies). Cells were then fixed with 4% formaldehyde (VWR 361387P) in PBS, permeablized with 0.1% Triton X-100 (Sigma-Aldrich X100) and blocked with 0.05% Triton X-100, 4% goat serum (Sigma-Aldrich G6767) and 1% bovine serum albumin (Sigma-Aldrich 9418) in PBS. HuHMFG1 was labelled with 0.098 mg/ml of anti-human IgG FITC (Sigma F5512), 0.05% Triton X-100 and 4% goat serum. Cells were then washed and co-stained with 300nM 4′,6-Diamidino-2-Phenylindole, Dilactate (DAPI) (Invitrogen D3571) in a mounting media made up from 0.5% N-Propyl gallate (Sigma-Aldrich P3130), 50% glycerol (VWR 24388.295) in 20mM Tris (Sigma-Aldrich T1503) pH 8. Images were collected at x63 magnification on a Perkin Elmer Spinning Disk Microscopy system using Volocity image acquisition software. FITC or Alexa Fluor 488 fluorescence was collected with excitation at 488nm and detection between 500 and 555nm and is shown in green. DAPI fluorescence was collected with excitation at 405nm and detection between 580 and 650nm and is shown in blue. PS1 or Cy5.5 fluorescence was collected with excitation at 640 nm and detection between 485-705 nm and is shown in red. Single stain control wells were included in the experiments and no bleed through was seen for any of the dyes/channels (data not shown).

### Production and characterization of HuHMFG1:PS1

N-Hydroxysuccinimide activated PS1 (PS1-NHS) was produced as previously published [41] and patented [42]. Small volume aliquots of PS1-NHS or a commercially available Cy5.5 NHS Ester (Amersham, GE Healthcare) were dissolved in DMSO were added progressively into a light protected PBS pH7.4 mixture containing 16.7μM HuHMFG1 and organic solvents at a final concentration of 20% DMSO and 6% MeCN, PS1-NHS or Cy5.5-NHS was added until 16 times molar excess over the protein. The reaction was left shaking and protected from light at room temperature for 2 hours. The resulting conjugates were dialyzed extensively into PBS pH 7.4 through a cellulose membrane with pore size MWCO 7kDa (Slide-A-Lyzer Dialysis Cassettes, 66370, Pierce), to remove unreacted or hydrolyzed PS1 as well as any organic solvents, neither gentle dialysis with a larger MWCO or size exclusion chromatography in the presence of low detergent concentrations were sufficient to improve the purity. For UV-VIS analyses a sample was diluted to a suitable concentration into PBS in a micro volume 1 cm path length quartz cuvette and absorbance was measured over 190-400nm on an Agilent 8453 UV Visible Spectrophotometer (Agilent Technologies). Spectra were normalized to 900 nm and PBS background removed. Concentrations were calculated using the following equation A = εlc where A is absorbance of the sample, ε = molar absorptivity, l = path length in cm and c = concentration in molar. Before spectra could be used to calculate conjugation efficiency, Molar extinction coefficient (M−1 cm−1) and the peak absorption were calculated for the free dyes in PBS and used as follows PS1 (A280=8896, A687=20594) and Cy5,5 (A280=22479, A674=215826), a generic IgG molar extinction coefficient was used for HuHMFG1 (A280=210000). To calculate conjugation ratio; absorbance of the conjugate at its peak absorbance in the red (678nm or 674nm) was used to obtain the concentration of the dye in the conjugate, this concentration could be used to calculate the contribution of the dye to absorption at 280nm. The remaining A280 is then attributed to the antibody and can be used to calculate the protein concentration. For reducing SDS analysis a sample of the conjugate was then denatured in reducing Laemmli Loading buffer that does not contain a loading dye, and run through on 12% Acrylamide SDS-PAGE. Fluorescence of the wet gels was visualized by exciting the photosensitizers with a UV-transilluminator (Fujifilm-LAS3000). Gels were then stained with Coomassie Brilliant Blue to visualize protein. Image analysis techniques of the unstained gel images were used to estimate the proportion of covalently coupled to free photosensitizer in the immunoconjugate mixture using AIDA image Analyzer software v3.52.

### Cytotoxicity studies

The cytotoxic efficacy of the HuHMFG1-PS1 ADC was compared with both equivalent concentrations of free HuHMFG1, and free equivalent PS1 in the presence or absence of laser “light” activation. MUC1 positive OE19 cells and MUC1 negative HT29 cells were plated in black walled 96 well plates and allowed to adhere over 24 hours. Cells were incubated at 37°C/5% CO_2_ in the dark with various doses of HuHMFG1, PS1 or HuHMFG1:PS1 ADC in supplemented cell culture media without phenol red (Sigma-Aldrich R7509) plus 2% DMSO. Controls included triton X 100 (100% cell death), culture media with 2% DMSO (vehicle control) and media alone with and without cells. After 2 hours cells were washed and returned to normal media and left at 37°C/5% CO_2_ for a further hour. Where cells were irradiated laser light was delivered at 670nm [0.33J/cm^2^ over 10seconds] (HPD 7401 laser system, High Power Devices Inc). Dark controls were not lasered. Cell viability was measured 48 hours later with MTS assay (Promega Cell Titre-96) via absorbance at 490nm. Background absorbance was removed using cells that had been lysed using triton X 100 and % cell viability was calculated using the cells in media only. Cytotoxicity between cell types was compared using the Students T-Test (Microsoft Excel). Dose response curves comparing cytotoxicity of HuHMFG1:PS1 ADC versus equivalent PS1 alone in light and dark were compared using linear regression analysis assigning best fit curves on a log scale (GraphPad PRISM ^®^).

### Transcript profiling

GEO accession numbers: GSE26886, GSE1420, GSE13083, GSE19529.

### Ethics

Ethical approval was obtained from the UK Research Ethics Committee (EC13.13; 08/H808/8; 08/H0714/27).

## SUPPLEMENTARY FIGURES



## Abbreviations

ADC: antibody drug conjugate
BE: Barrett’s epithelium
EA: esophageal adenocarcinoma
FFPE: formalin fixed paraffin embedded
GSEA: gene set enrichment analysis
H&E: haematoxylin and eosin
HER2: human epidermal growth factor receptor 2
HGD: high grade dysplasia
IHC: immunohistochemistry
LGD: low grade dysplasia
MUC1: mucin glycoprotein 1
NDBE: non-dysplastic Barrett’s esophagus
PBS: phosphate-buffered saline
PDT: photodynamic therapy
PS: photosensitizer
SDS-PAGE: sodium dodecyl sulfate polyacrylamide gel electrophoresis
Sq: squamous
UV-Vis: ultraviolet–visible spectroscopy
VNTR: variable number tandem repeat

## References

[1] Cancer Research UK Cancer Survival Group. (2014). Age-Standardised One-, Five- and Ten-Year Net Survival, Adults (Aged 15-99), England and Wales.

[2] Sharma P, Falk GW, Sampliner R, Jon Spechler S, Wang K (2009). Management of nondysplastic Barrett’s esophagus: where are we now?. Am J Gastroenterol.

[3] Solaymani-Dodaran M, Logan RFA, West J, Card T (2005). Mortality associated with Barrett’s esophagus and gastroesophageal reflux disease diagnoses-a population-based cohort study. Am J Gastroenterol.

[4] Haidry RJ, Dunn JM, Butt MA, Burnell MG, Gupta A, Green S, Miah H, Smart HL, Bhandari P, Smith LA, Willert R, Fullarton G, Morris J (2013). Radiofrequency ablation and endoscopic mucosal resection for dysplastic Barrett’s esophagus and early esophageal adenocarcinoma: Outcomes of the UK national halo RFA registry. Gastroenterology.

[5] Bang YJ, Van Cutsem E, Feyereislova A, Chung HC, Shen L, Sawaki A, Lordick F, Ohtsu A, Omuro Y, Satoh T, Aprile G, Kulikov E, Hill J (2010). Trastuzumab in combination with chemotherapy versus chemotherapy alone for treatment of HER2-positive advanced gastric or gastro-oesophageal junction cancer (ToGA): A phase 3, open-label, randomised controlled trial. Lancet.

[6] Yoon HH, Shi Q, Sukov WR, Lewis MA, Sattler CA, Wiktor AE, Wu T-T, Diasio RB, Jenkins RB, Sinicrope FA (2012). Adverse prognostic impact of intratumor heterogeneous HER2 gene amplification in patients with esophageal adenocarcinoma. J Clin Oncol.

[7] Stahl P, Seeschaaf C, Lebok P, Kutup A, Bockhorn M, Izbicki JR, Bokemeyer C, Simon R, Sauter G, Marx AH (2015). Heterogeneity of amplification of HER2, EGFR, CCND1 and MYC in gastric cancer. BMC Gastroenterol.

[8] Nath S, Mukherjee P (2014). MUC1: A multifaceted oncoprotein with a key role in cancer progression. Trends Mol Med.

[9] Bafna S, Kaur S, Batra SK (2010). Membrane-bound mucins: the mechanistic basis for alterations in the growth and survival of cancer cells. Oncogene.

[10] Yonezawa S, Goto M, Yamada N, Higashi M, Nomoto M (2008). Expression profiles of MUC1, MUC2, and MUC4 mucins in human neoplasms and their relationship with biological behavior. Proteomics.

[11] Piessen G, Wacrenier a, Briez N, Triboulet J-P, Van Seuningen I, Mariette C (2009). Clinical impact of MUC1 and MUC4 expression in Barrett-associated oesophageal adenocarcinoma. J Clin Pathol.

[12] Arul GS, Moorghen M, Myerscough N, Alderson D a, Spicer RD (2000). Corfield a P. Mucin gene expression in Barrett’s oesophagus: an in situ hybridisation and immunohistochemical study. Gut.

[13] Guillem P, Billeret V, Buisine MP, Flejou JF, Lecomte-Houcke M, Degand P, Aubert JP, Triboulet JP, Porchet N (2000). Mucin gene expression and cell differentiation in human normal, premalignant and malignant esophagus. Int J Cancer.

[14] Mariette C, Piessen G, Leteurtre E, Hémon B, Triboulet JP, Van Seuningen I (2008). Activation of MUC1 mucin expression by bile acids in human esophageal adenocarcinomatous cells and tissues is mediated by the phosphatidylinositol 3-kinase. Surgery.

[15] Palmer AJ, Lochhead P, Hold GL, Rabkin CS, Chow W-H, Lissowska J, Vaughan TL, Berry S, Gammon M, Risch H, El-Omar EM (2012). Genetic variation in C20orf54, PLCE1 and MUC1 and the risk of upper gastrointestinal cancers in Caucasian populations. Eur J Cancer Prev.

[16] Pegram MD, Borges VF, Ibrahim N, Fuloria J, Shapiro C, Perez S, Wang K, Schaedli Stark F, Courtenay Luck N (2009). Phase I dose escalation pharmacokinetic assessment of intravenous humanized anti-MUC1 antibody AS1402 in patients with advanced breast cancer. Breast Cancer Res.

[17] Royer B, Yin W, Pegram M, Ibrahim N, Villanueva C, Mir D, Erlandsson F, Pivot X (2010). Population pharmacokinetics of the humanised monoclonal antibody, HuHMFG1 (AS1402), derived from a phase I study on breast cancer. Br J Cancer.

[18] Ibrahim NK, Yariz KO, Bondarenko I, Manikhas A, Semiglazov V, Alyasova A, Komisarenko V, Shparyk Y, Murray JL, Jones D, Senderovich S, Chau A, Erlandsson F (2011). Randomized phase II trial of letrozole plus Anti-MUC1 antibody AS1402 in hormone receptor-positive locally advanced or metastatic breast cancer. Clin Cancer Res.

[19] Nicholson S, Bomphray CC, Thomas H, McIndoe A, Barton D, Gore M, George AJT (2004). A phase I trial of idiotypic vaccination with HMFG1 in ovarian cancer. Cancer immunology, immunotherapy: CII.

[20] Oei ALM, Moreno M, Verheijen RHM, Sweep FCGJ, Thomas CMG, Massuger LFAG, von Mensdorff-Pouilly S (2008). Induction of IgG antibodies to MUC1 and survival in patients with epithelial ovarian cancer. International journal of cancer. Journal international du cancer.

[21] Pericleous LM, Richards J, Epenetos a a, Courtenay-Luck N, Deonarain MP (2005). Characterisation and internalisation of recombinant humanised HMFG-1 antibodies against MUC1. Br J Cancer.

[22] Hamann PR, Hinman LM, Beyer CF, Lindh D, Upeslacis J, Shochat D, Mountain A (2005). A calicheamicin conjugate with a fully humanized anti-MUC1 antibody shows potent antitumor effects in breast and ovarian tumor xenografts. Bioconjug Chem.

[23] Overholt BF, Wang KK, Burdick JS, Lightdale CJ, Kimmey M, Nava HR, Sivak M V., Nishioka N, Barr H, Marcon N, Pedrosa M, Bronner MP, Grace M (2007). Five-year efficacy and safety of photodynamic therapy with Photofrin in Barrett’s high-grade dysplasia. Gastrointest Endosc.

[24] Oniszczuk A, Wojtunik-Kulesza KA, Oniszczuk T, Kasprzak K (2016). The potential of photodynamic therapy (PDT)-Experimental investigations and clinical use. Biomed Pharmacother.

[25] Bown SG (2012). How mainstream medicine sees photodynamic therapy in the United Kingdom. J Natl Compr Canc Netw.

[26] Castano AP, Demidova TN, Hamblin MR (2005). Mechanisms in photodynamic therapy: Part two - Cellular signaling, cell metabolism and modes of cell death. Photodiagnosis Photodyn Ther.

[27] Castano AP, Demidova TN, Hamblin MR (2004). Mechanisms in photodynamic therapy: Part one - Photosensitizers, photochemistry and cellular localization. Photodiagnosis Photodyn Ther.

[28] Castano AP, Demidova TN, Hamblin MR (2005). Mechanisms in photodynamic therapy: Part three - Photosensitizer pharmacokinetics, biodistribution, tumor localization and modes of tumor destruction. Photodiagnosis Photodyn Ther.

[29] Bown SG, Lovat LB (2000). The biology of photodynamic therapy in the gastrointestinal tract. Gastrointest Endosc Clin N Am.

[30] Mew D, Wat CK, Towers GH, Levy JG (1983). Photoimmunotherapy: treatment of animal tumors with tumor-specific monoclonal antibody-hematoporphyrin conjugates. J Immunol.

[31] Hemming AW, Davis NL, Dubois B, Quenville NF, Finley RJ (1993). Photodynamic therapy of squamous cell carcinoma. An evaluation of a new photosensitizing agent, benzoporphyrin derivative and new photoimmunoconjugate. Surg Oncol.

[32] Hamblin MR, Del Governatore M, Rizvi I, Hasan T (2000). Biodistribution of charged 17.1A photoimmunoconjugates in a murine model of hepatic metastasis of colorectal cancer. Br J Cancer.

[33] Bhatti M, Yahioglu G, Milgrom LR, Garcia-Maya M, Chester K a., Deonarain MP (2008). Targeted photodynamic therapy with multiply-loaded recombinant antibody fragments. Int J Cancer.

[34] Jiang FN, Jiang S, Liu D, Richter A, Levy JG (1990). Development of technology for linking photosensitizers to a model monoclonal antibody. J Immunol Methods.

[35] Mitsunaga M, Ogawa M, Kosaka N, Rosenblum LT, Choyke PL, Kobayashi H (2011). Cancer cell–selective in vivo near infrared photoimmunotherapy targeting specific membrane molecules. Nat Med.

[36] Palumbo a, Hauler F, Dziunycz P, Schwager K, Soltermann a, Pretto F, Alonso C, Hofbauer GF, Boyle RW, Neri D (2011). A chemically modified antibody mediates complete eradication of tumours by selective disruption of tumour blood vessels. Br J Cancer.

[37] Hayley P, Stamati I, Yahioglu G, Butt M, Deonarain M (2013). Antibody-Directed Phototherapy (ADP). Antibodies.

[38] Savellano MD, Owusu-Brackett N, Son J, Ganga T, Leung NL, Savellano DH Photodynamic tumor eradication with a novel targetable photosensitizer: strong vascular effects and dependence on treatment repetition versus potentiation. Photochem Photobiol.

[39] Fitzgerald RC, di Pietro M, Ragunath K, Ang Y, Kang JY, Watson P, Trudgill N, Patel P, Kaye P V, Sanders S, O’Donovan M, Bird-Lieberman E, Bhandari P (2014). British Society of Gastroenterology guidelines on the diagnosis and management of Barrett’s oesophagus. Gut.

[40] Berenson MM, Johnson TD, Markowitz NR, Buchi KN, Samowitz WS (1993). Restoration of squamous mucosa after ablation of Barrett’s esophageal epithelium. Gastroenterology.

[41] Stamati I, Kuimova MK, Lion M, Yahioglu G, Phillips D, Deonarain MP (2010). Novel photosensitisers derived from pyropheophorbide-a: uptake by cells and photodynamic efficiency in vitro. Photochem Photobiol Sci.

[42] Yahioglu G, Stamati I, Deonarain M (2010). Compounds and biological materials and uses thereof. WO 2010106341 A1.

[43] Sagara M, Yonezawa S, Nagata K, Tezuka Y, Natsugoe S, Xing PX, Mckenzie IFC, Aikou T, Sato E (1999). Expression of mucin 1 (MUC1) in esophageal squamous-cell carcinoma: Its relationship with prognosis. Int J Cancer.

[44] Lloyd KO, Burchell J, Yin BWT, Kudryashov V, Taylor-papadimitriou J (1996). Products: Comparison of O -Linked Carbohydrate Chains in MUC-1 Mucin from Normal Breast Epithelial Cell Lines and Breast Carcinoma Cell Lines: FEWER GLYCAN CHAINS IN TUMOR Comparison of O -Linked Carbohydrate Chains in MUC-1 Mucin from Normal Breast Ep.

[45] Ikeda Y, Kuwano H, Baba K, Ikebe M, Matushima T, Adachi Y, Mori M, Sugimachi K (1993). Expression of Sialyl-Tn antigens in normal squamous epithelium, dysplasia, and squamous cell carcinoma in the esophagus. Cancer Res.

[46] Fowler JC, Teixeira AS, Vinall LE, Swallow DM (2003). Hypervariability of the membrane-associated mucin and cancer marker MUC1. Hum Genet.

[47] Dunn JM, Mackenzie GD, Banks MR, Mosse CA, Haidry R, Green S, Thorpe S, Rodriguez-Justo M, Winstanley A, Novelli MR, Bown SG, Lovat LB (2013). A randomised controlled trial of ALA vs. Photofrin photodynamic therapy for high-grade dysplasia arising in Barrett’s oesophagus. Lasers Med Sci.

[48] Bolin FP, Preuss LE, Taylor RC (1987). Optimization of photodynamic therapy light dose distribution and treatment volume by multi-fiber insertions. Photochem Photobiol.

[49] Shackley DC, Whitehurst C, Moore J V, George NJ, Betts CD, Clarke NW (2000). Light penetration in bladder tissue: implications for the intravesical photodynamic therapy of bladder tumours. BJU Int.

[50] Savellano MD, Hasan T (2003). Targeting cells that overexpress the epidermal growth factor receptor with polyethylene glycolated BPD verteporfin photosensitizer immunoconjugates. Photochem Photobiol.

[51] Pye H, Butt MA, Reinert HW, Maruani A, Nunes JPM, Marklew JS, Qurashi M, Funnell L, May A, Stamati I, Hamoudi R, Baker JR, Smith MEB (2016). A HER2 selective theranostic agent for surgical resection guidance and photodynamic therapy. Photochem Photobiol Sci.

[52] Wang Q, Ma C, Kemmner W (2013). Wdr66 is a novel marker for risk stratification and involved in epithelial-mesenchymal transition of esophageal squamous cell carcinoma. BMC Cancer.

[53] Hamoudi RA, Appert A, Ye H, Ruskone-Fourmestraux A, Streubel B, Chott A, Raderer M, Gong L, Wlodarska I, De Wolf-Peeters C, MacLennan KA, de Leval L, Isaacson PG (2010). Differential expression of NF-kappaB target genes in MALT lymphoma with and without chromosome translocation: insights into molecular mechanism. Leukemia.

[54] Kimchi ET, Posner MC, Park JO, Darga TE, Kocherginsky M, Karrison T, Hart J, Smith KD, Mezhir JJ, Weichselbaum RR, Khodarev NN (2005). Progression of Barrett’s metaplasia to adenocarcinoma is associated with the suppression of the transcriptional programs of epidermal differentiation. Cancer Res.

[55] Stairs DB, Nakagawa H, Klein-Szanto A, Mitchell SD, Silberg DG, Tobias JW, Lynch JP, Rustgi AK (2008). Cdx1 and c-Myc foster the initiation of transdifferentiation of the normal esophageal squamous epithelium toward Barrett’s esophagus. PLoS One.

[56] Saadi A, Shannon NB, Lao-Sirieix P, O’Donovan M, Walker E, Clemons NJ, Hardwick JS, Zhang C, Das M, Save V, Novelli M, Balkwill F, Fitzgerald RC (2010). Stromal genes discriminate preinvasive from invasive disease, predict outcome, and highlight inflammatory pathways in digestive cancers. Proc Natl Acad Sci U S A.

[57] Rakha EA, Boyce RWG, Abd El-Rehim D, Kurien T, Green AR, Paish EC, Robertson JFR, Ellis IO (2005). Expression of mucins (MUC1, MUC2, MUC3, MUC4, MUC5AC and MUC6) and their prognostic significance in human breast cancer. Mod Pathol.

[58] Gendler S, Taylor-Papadimitriou J, Duhig T, Rothbard J, Burchell J (1988). A highly immunogenic region of a human polymorphic epithelial mucin expressed by carcinomas is made up of tandem repeats. J Biol Chem.

[59] Pemberton L, Taylor-Papadimitriou J, Gendler SJ (1992). Antibodies to the cytoplasmic domain of the MUC1 mucin show conservation throughout mammals. Biochem Biophys Res Commun.

[60] Spencer DIR, Price MR, Tendler SJB, De Matteis CI, Stadie T, Hanisch FG (1996). Effect of glycosylation of a synthetic MUC1 mucin-core-related peptide on recognition by anti-mucin antibodies. Cancer Lett.

[61] Burchell J, Taylor-Papadimitriou J (1993). Effect of modification of carbohydrate side chains on the reactivity of antibodies with core-protein epitopes of the MUC1 gene product. Epithelial Cell Biol.

[62] Phillips T, Murray G, Wakamiya K, Askaa J, Huang D, Welcher R, Pii K, Allred DC (2007). Development of standard estrogen and progesterone receptor immunohistochemical assays for selection of patients for antihormonal therapy. Appl Immunohistochem Mol Morphol.

[63] Stoner GD, Kaighn ME, Reddel RR, Resau JH, Bowman D, Naito Z, Matsukura N, You M, Galati AJ, Harris CC (1991). Establishment and characterization of SV40 T-antigen immortalized human esophageal epithelial cells. Cancer Res.

[64] Underwood TJ, Derouet MF, White MJ, Noble F, Moutasim K a, Smith E, Drew P a, Thomas GJ, Primrose JN, Blaydes JP (2010). A comparison of primary oesophageal squamous epithelial cells with HET-1A in organotypic culture. Biol Cell.

[65] Jaiswal KR, Morales CP, Feagins L a., Gandia KG, Zhang X, Zhang HY, Hormi-Carver K, Shen Y, Elder F, Ramirez RD, Sarosi G a., Spechler SJ, Souza RF (2007). Characterization of telomerase-immortalized, non-neoplastic, human Barrett’s cell line (BAR-T). Dis Esophagus.

[66] Palanca-Wessels MC, Barrett MT, Galipeau PC, Rohrer KL, Reid BJ, Rabinovitch PS (1998). Genetic analysis of long-term Barrett’s esophagus epithelial cultures exhibiting cytogenetic and ploidy abnormalities. Gastroenterology.

[67] Rockett JC, Larkin K, Darnton SJ, Morris a G, Matthews HR (1997). Five newly established oesophageal carcinoma cell lines: phenotypic and immunological characterization. Br J Cancer.

