# Supplementary material for: Upregulation of mucin glycoprotein MUC1 in the progression to esophageal adenocarcinoma and therapeutic potential with a targeted photoactive antibody-drug conjugate
Source: Oncotarget. 2017 Feb 15;8(15):25080–96. doi: 10.18632/oncotarget.15340 (PMC5421911; doi:10.18632/oncotarget.15340)
Supplement: Supplementary file 1 [file oncotarget-08-25080-s001.pdf]

Upregulation of mucin glycoprotein MUC1 in the progression to esophageal adenocarcinoma and therapeutic potential with a targeted photoactive antibody-drug conjugate

SUPPLEMENTARY FIGURES

A

Gene Set	Contains MUC1	SIZE	Enrichment Score (ES)	Normalised ES	Nominal p-value	False Discovery Rate (FDR) q-val	FWER p-val	Tag %	Gene %	Signal	FDR (median)	glob. p.val
BRCA_ER_POS	YES	334	0.38703	1.7166	0	0.032246	0.04527	0.371	0.292	0.272	0	0.01
POST_TRANSLATIONAL_PROTEIN_MODIFICATION	NO	273	0.31988	1.6258	0	0.023622	0.1076	0.516	0.47	0.282	0	0
PROTEIN_MODIFICATION_PROCESS	NO	354	0.32404	1.6101	0	0.022916	0.1217	0.517	0.47	0.285	0	0
CELL_PROLIFERATION_GO_0008283	NO	280	0.34251	1.5959	0	0.024012	0.1348	0.5	0.447	0.285	0	0
REGULATION_OF_CATALYTIC_ACTIVITY	NO	161	0.31555	1.5358	0	0.02607	0.2183	0.578	0.524	0.28	0	0
REGULATION_OF_MOLECULAR_FUNCTION	NO	181	0.30988	1.5256	0	0.027405	0.2354	0.453	0.423	0.266	0	0
HSC_HSCANDPROGENITORS_ADULT	YES	353	0.31448	1.6321	0.001976	0.025468	0.1056	0.227	0.207	0.186	0	0
PROLIFERATION_GENES	NO	207	0.37767	1.5887	0.002008	0.024533	0.1459	0.512	0.421	0.303	0	0
TRANSMEMBRANE_RECEPTOR_PROTEIN_TYROSINE_KINASE_SIGNALING_PATHWAY	NO	47	0.41241	1.6706	0.002066	0.03709	0.08249	0.404	0.289	0.289	0	0.008
PHOSPHORYLATION	NO	178	0.30448	1.4936	0.003717	0.029863	0.2706	0.517	0.469	0.279	0	0
HSC_HSCANDPROGENITORS_FETAL	YES	345	0.29927	1.5647	0.003945	0.020216	0.17	0.214	0.207	0.176	0	0
CELL_SURFACE_RECEPTOR_LINKED_SIGNAL_TRANSDUCTION_GO_0007166	NO	297	0.37034	1.5846	0.004024	0.023231	0.1479	0.444	0.372	0.288	0	0
REGULATION_OF_CELL_PROLIFERATION	NO	166	0.37084	1.5742	0.004065	0.019073	0.1559	0.494	0.42	0.292	0	0
REGULATION_OF_PROTEIN_KINASE_ACTIVITY	NO	100	0.36053	1.6456	0.005837	0.025088	0.09457	0.46	0.387	0.285	0	0
REGULATION_OF_KINASE_ACTIVITY	NO	101	0.36433	1.6588	0.005871	0.029898	0.08954	0.465	0.387	0.288	0	0.003
REGULATION_OF_KINASE_ACTIVITY	NO	101	0.36433	1.6588	0.005871	0.029898	0.08954	0.465	0.387	0.288	0	0.003
REGULATION_OF_MAP_KINASE_ACTIVITY	NO	45	0.44114	1.6918	0.006024	0.034542	0.06338	0.6	0.387	0.369	0	0.008
ENZYME_LINKED_RECEPTOR_PROTEIN_SIGNALING_PATHWAY	NO	84	0.37688	1.577	0.006316	0.019792	0.1549	0.452	0.368	0.288	0	0
PROTEIN_AMINO_ACID_PHOSPHORYLATION	NO	163	0.31151	1.4874	0.007634	0.029468	0.2817	0.521	0.469	0.281	0	0
REGULATION_OF_TRANSFERASE_ACTIVITY	NO	102	0.36706	1.6585	0.007921	0.026326	0.09054	0.471	0.387	0.291	0	0
POSITIVE_REGULATION_OF_MAP_KINASE_ACTIVITY	NO	28	0.47933	1.5825	0.01022	0.022252	0.1489	0.607	0.366	0.386	0	0
LEI_MYB_REGULATED_GENES	YES	218	0.41855	1.6212	0.01597	0.022352	0.1097	0.541	0.391	0.337	0	0
CELL_PROLIFERATION	NO	118	0.3645	1.5775	0.018	0.020679	0.1549	0.559	0.451	0.311	0	0
SHEPARD_CELL_PROLIFERATION	NO	118	0.3645	1.5775	0.018	0.020679	0.1549	0.559	0.451	0.311	0	0
CELL_GROWTH_AND_OR_MAINTENANCE	NO	39	0.44008	1.496	0.02	0.030252	0.2656	0.487	0.347	0.32	0	0
TPA_SENS_MIDDLE_DN	YES	187	0.31567	1.4612	0.02037	0.035419	0.326	0.54	0.494	0.278	0	0
FRASOR_ER_DN	NO	47	0.40692	1.4932	0.02204	0.029019	0.2716	0.553	0.394	0.337	0	0
BREAST_CANCER_ESTROGEN_SIGNALING	YES	63	0.41189	1.5035	0.04225	0.029637	0.2515	0.365	0.262	0.271	0	0
CIS_XPC_UP	YES	98	0.36627	1.5002	0.05758	0.029279	0.2555	0.306	0.27	0.226	0	0
HUMAN_TISSUE_PANCREAS	YES	24	0.40599	1.3091	0.09393	0.10168	0.5875	0.25	0.193	0.202	0.054994	0
TPA_RESIST_EARLY_DN	YES	50	0.35588	1.2717	0.1113	0.12427	0.6398	0.16	0.0938	0.146	0.072983	0
MITOCHONDRIA	NO	183	0.25868	1.2262	0.1717	0.15843	0.7103	0.568	0.551	0.26	0.105	0.001
TPA_RESIST_MIDDLE_DN	YES	70	0.30725	1.1826	0.1738	0.1856	0.7787	0.514	0.506	0.256	0.13462	0.001
FERNANDEZ_MYC_TARGETS	YES	94	0.31121	1.2226	0.1814	0.15796	0.7163	0.574	0.506	0.286	0.1047	0
BRCA1_OVEREXP_PROSTATE_UP	YES	117	0.30584	1.2071	0.1835	0.16934	0.7435	0.65	0.56	0.289	0.12039	0.001
HSA04012_ERBB_SIGNALING_PATHWAY	NO	53	0.28765	1.178	0.2037	0.18444	0.7797	0.472	0.46	0.256	0.13611	0
LINDSTEDT_DEND_8H_VS_48H_DN	YES	51	0.34768	1.2028	0.2153	0.16896	0.7475	0.667	0.512	0.327	0.11722	0.001
BREASTCA_TWO_CLASSES	NO	83	0.29425	1.1702	0.2484	0.18856	0.7867	0.386	0.379	0.241	0.14285	0
DRUG_RESISTANCE_AND_METABOLISM	NO	61	0.31168	1.1463	0.278	0.20944	0.8139	0.508	0.484	0.264	0.16204	0
NFkBinduc_w_MUC1	YES	161	0.314	1.1148	0.2992	0.23881	0.8431	0.255	0.229	0.2	0.18992	0
BROCKE_IL6	YES	106	0.2655	1.0985	0.3314	0.24917	0.8622	0.406	0.442	0.229	0.2084	0
KRETZSCHMAR_IL6_DIFF	YES	106	0.2655	1.0985	0.3314	0.24917	0.8622	0.406	0.442	0.229	0.2084	0
KLEIN_PEL_UP	YES	33	0.29514	1.0633	0.3397	0.28796	0.8833	0.727	0.589	0.3	0.25946	0
HYPOXIA_REG_UP	YES	27	0.33519	0.93025	0.5752	0.52142	0.9678	0.741	0.559	0.327	0.52128	0.004
CHAUHAN_2ME2	YES	30	0.29025	0.90195	0.6055	0.56545	0.9688	0.533	0.528	0.252	0.58333	0.006
RADIATION_SENSITIVITY	NO	15	0.26098	0.77633	0.7752	0.80357	0.9889	0.267	0.326	0.18	0.8524	0.062

B

Gene Set	Contains MUC1	SIZE	Enrichment Score (ES)	Normalised ES	Nominal p-value	False Discovery Rate (FDR) q-val	FWER p-val	Tag %	Gene %	Signal	FDR (median)	glob. p.val
TPA_RESIST_EARLY_DN	YES	52	0.42742	1.6617	0	0.031003	0.1041	0.269	0.169	0.225	0	0.003
PROTEIN_MODIFICATION_PROCESS	NO	388	0.30516	1.5859	0	0.045142	0.1702	0.309	0.293	0.227	0	0.003
FERNANDEZ_MYC_TARGETS	YES	109	0.39083	1.7664	0	0.015905	0.04404	0.44	0.347	0.291	0	0.002
TRANSMEMBRANE_RECEPTOR_PROTEIN_TYROSINE_KINASE_SIGNALING_PATHWAY	NO	48	0.49821	1.9249	0	0.0038311	0.005005	0.458	0.287	0.328	0	0.001
ENZYME_LINKED_RECEPTOR_PROTEIN_SIGNALING_PATHWAY	NO	87	0.43756	1.8645	0	0.0084891	0.01502	0.356	0.227	0.278	0	0.001
BRCA_ER_POS	YES	377	0.37525	1.7535	0	0.013113	0.04905	0.366	0.302	0.265	0	0.001
POST_TRANSLATIONAL_PROTEIN_MODIFICATION	NO	294	0.29568	1.4925	0	0.042743	0.3073	0.306	0.293	0.222	0	0.001
REGULATION_OF_MAP_KINASE_ACTIVITY	NO	44	0.46894	1.7623	0.001905	0.013432	0.04505	0.477	0.287	0.341	0	0.001
LEI_MYB_REGULATED_GENES	YES	240	0.43097	1.7861	0.002141	0.015645	0.03403	0.454	0.315	0.318	0	0.004
REGULATION_OF_PROTEIN_KINASE_ACTIVITY	NO	101	0.37146	1.5852	0.005714	0.041711	0.1722	0.426	0.322	0.291	0	0.003
HSC_HSCANDPROGENITORS_FETAL	YES	397	0.30164	1.521	0.005859	0.040204	0.2613	0.338	0.336	0.233	0	0
HSC_HSCANDPROGENITORS_ADULT	YES	408	0.30283	1.5266	0.005871	0.040631	0.2502	0.336	0.333	0.233	0	0.001
REGULATION_OF_CELL_PROLIFERATION	NO	184	0.37611	1.6016	0.005952	0.045447	0.1612	0.413	0.329	0.282	0	0.005
REGULATION_OF_KINASE_ACTIVITY	NO	102	0.36812	1.5612	0.007678	0.042096	0.1972	0.422	0.322	0.289	0	0.002
REGULATION_OF_KINASE_ACTIVITY	NO	102	0.36812	1.5612	0.007678	0.042096	0.1972	0.422	0.322	0.289	0	0.002
REGULATION_OF_CATALYTIC_ACTIVITY	NO	165	0.32708	1.5335	0.009881	0.042522	0.2402	0.388	0.345	0.258	0	0.001
REGULATION_OF_TRANSFERASE_ACTIVITY	NO	103	0.36712	1.556	0.01163	0.038658	0.2022	0.417	0.322	0.286	0	0.001
BRCA1_OVEREXP_PROSTATE_UP	YES	122	0.38177	1.5293	0.012	0.042042	0.2482	0.443	0.365	0.284	0	0.001
POSITIVE_REGULATION_OF_MAP_KINASE_ACTIVITY	NO	28	0.48034	1.6274	0.01426	0.037837	0.1361	0.464	0.264	0.342	0	0.002
CELL_PROLIFERATION_GO_0008283	NO	307	0.34584	1.5002	0.01765	0.045728	0.2893	0.388	0.324	0.27	0	0.002
CELL_GROWTH_AND_OR_MAINTENANCE	NO	46	0.46253	1.5573	0.02209	0.040646	0.2012	0.326	0.174	0.271	0	0.002
CELL_SURFACE_RECEPTOR_LINKED_SIGNAL_TRANSDUCTION_GO_0007166	NO	323	0.37928	1.5372	0.02231	0.044058	0.2372	0.35	0.287	0.257	0	0.001
REGULATION_OF_MOLECULAR_FUNCTION	NO	184	0.30918	1.4265	0.02245	0.054469	0.3944	0.375	0.345	0.25	0	0
PROLIFERATION_GENES	NO	229	0.35774	1.4399	0.02677	0.052864	0.3784	0.393	0.332	0.268	0	0
CELL_PROLIFERATION	NO	128	0.39426	1.4968	0.0334	0.042917	0.2953	0.398	0.296	0.284	0	0.001
SHEPARD_CELL_PROLIFERATION	NO	128	0.39426	1.4968	0.0334	0.042917	0.2953	0.398	0.296	0.284	0	0.001
KLEIN_PEL_UP	YES	34	0.40858	1.4709	0.04008	0.044725	0.3303	0.412	0.333	0.275	0	0
DRUG_RESISTANCE_AND_METABOLISM	NO	56	0.39119	1.4288	0.04483	0.055736	0.3934	0.429	0.318	0.294	0	0.001
TPA_SENS_MIDDLE_DN	YES	217	0.30057	1.3753	0.054	0.067614	0.4965	0.41	0.394	0.253	0	0
PHOSPHORYLATION	NO	193	0.27325	1.3428	0.05697	0.078203	0.5495	0.601	0.565	0.266	0.037926	0
POSITIVE_REGULATION_OF_TRANSFERASE_ACTIVITY	NO	52	0.37675	1.4523	0.06012	0.049866	0.3644	0.404	0.322	0.275	0	0
BREAST_CANCER_ESTROGEN_SIGNALING	YES	69	0.37944	1.4049	0.07292	0.058909	0.4424	0.507	0.42	0.296	0	0
BREAST_CANCER_ESTROGEN_SIGNALING	YES	69	0.37944	1.4049	0.07292	0.058909	0.4424	0.507	0.42	0.296	0	0
HSA04012_ERBB_SIGNALING_PATHWAY	NO	53	0.34618	1.3503	0.07407	0.076464	0.5385	0.396	0.339	0.263	0.037838	0
HUMAN_TISSUE_PANCREAS	YES	25	0.4677	1.3793	0.08119	0.06806	0.4925	0.32	0.156	0.271	0	0
FRASOR_ER_DN	NO	56	0.41148	1.405	0.08317	0.062591	0.4424	0.554	0.402	0.333	0	0
NFkBinduc_w_MUC1	YES	177	0.42197	1.468	0.08583	0.046157	0.3373	0.322	0.223	0.254	0	0
PROTEIN_AMINO_ACID_PHOSPHORYLATION	NO	174	0.2653	1.2565	0.1277	0.12926	0.6757	0.598	0.565	0.264	0.083761	0.001
MITOCHONDRIA	NO	193	0.24052	1.203	0.1646	0.16388	0.7638	0.528	0.554	0.24	0.12017	0.001
TPA_RESIST_MIDDLE_DN	YES	78	0.30059	1.1966	0.1798	0.16615	0.7728	0.167	0.13	0.146	0.12661	0
BROCKE_IL6	YES	120	0.30288	1.2202	0.2045	0.15131	0.7297	0.417	0.404	0.251	0.10865	0.001
KRETZSCHMAR_IL6_DIFF	YES	120	0.30288	1.2202	0.2045	0.15131	0.7297	0.417	0.404	0.251	0.10865	0.001
CIS_XPC_UP	YES	109	0.30026	1.184	0.2227	0.17448	0.7898	0.349	0.333	0.235	0.13364	0
BREASTCA_TWO_CLASSES	NO	90	0.26542	1.1385	0.2724	0.21068	0.8218	0.322	0.362	0.207	0.17376	0
HYPOXIA_REG_UP	YES	32	0.374	1.1776	0.2843	0.17691	0.7968	0.625	0.477	0.328	0.13611	0
LINDSTEDT_DEND_8H_VS_48H_DN	YES	53	0.35692	1.1745	0.286	0.17601	0.7968	0.434	0.417	0.254	0.13701	0
CHAUHAN_2ME2	YES	33	0.24302	0.89983	0.6198	0.56762	0.986	0.636	0.625	0.239	0.57939	0.008
RADIATION_SENSITIVITY	NO	16	0.21347	0.63316	0.8934	0.96488	0.998	0.375	0.437	0.212	1	0.606

Supplementary Figure 1: Gene set enrichment analysis highlighting pathways during development of Barrett's esophagus and esophageal cancer and the involvement of MUC1 within them. In the gene set enrichment assay (GSEA) two groups of esophageal tissue were compared. Significant pathways are highlighted in blue text (nominal p-value <0.05) and those containing MUC1 highlighted with orange background. **A.** The comparison of non-dysplastic Barrett's esophagus (NDBE) to normal esophageal squamous epithelium gave 27 pathways significantly enriched in NDBE compared to squamous epithelium. **B.** Comparison of esophageal adenocarcinoma (EA) to normal esophageal squamous epithelium gave 28 pathways significantly enriched in EA. Many of the significant pathways contained MUC1 including the most significantly enriched pathway in each pairing.

A

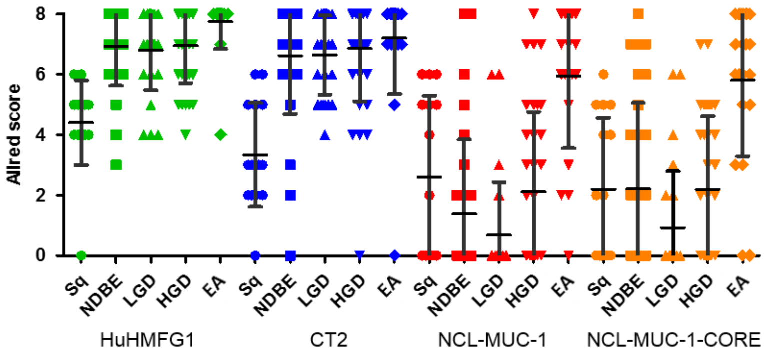

B

One-way analysis of variance

	HuHMFG1	CT2	NCL-MUC-1	NCL- MUC-1-CORE
P value	< 0.0001	< 0.0001	< 0.0001	< 0.0001
F	15.07	15.01	13.55	13.27

Post-test for linear trend

	HuHMFG1	CT2	NCL-MUC-1	NCL- MUC-1-CORE
Slope	0.5386	0.8451	0.823	0.8293
P value	< 0.0001	< 0.0001	< 0.0001	< 0.0001

Supplementary Figure 2: Scatter plot depicting immunohistochemical expression of four MUC1 epitopes in the esophageal squamous-metaplasia-dysplasia-carcinoma sequence scored by Allred. A. Immunohistochemical expression of anti-MUC1 antibodies HuHMFG1, CT2, NCL-MUC-1 and NCL-MUC-1-CORE were evaluated with the Allred score in normal squamous epithelium (Sq), non-dysplastic Barrett's esophagus (NDBE), low-grade dysplasia (LGD), high grade dysplasia (HGD) and invasive esophageal adenocarcinoma (EA). The Allred score (0- to 8) combines the proportion of cells that stain positive (on a scale of 0 to 5) with staining intensity (on a scale of 0 to 3). Allred is plotted for each sample with mean and standard deviation. **B.** All antibodies show a statistically significant incremental increase in positivity during progression to cancer.

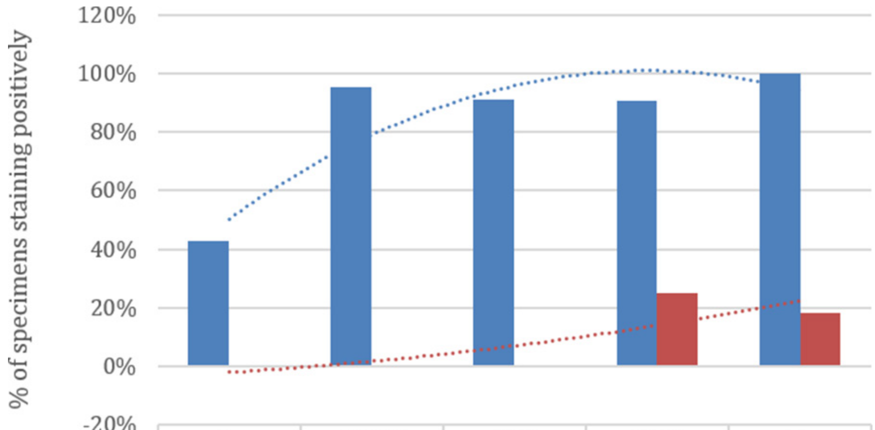

	Sq	NDBE	LGD	HGD	EA
1 in 1000	43%	95%	91%	91%	100%
1 in 10,000	0.00%	0.00%	0.00%	25.00%	18.18%

Supplementary Figure 3: HuHMFG1 immunostaining optimized to achieve tumor selectivity. HuHMFG1 antibody concentration was re-optimized to maintain positivity (2+/3+ intensity immunostaining) in higher grade pathologies, whilst excluding normal squamous (Sq) positivity. This concentration was found to be 10x fold less than that used in the original experiment (1:1000 vs 1:10,000). Specificity was achieved for high grade dysplasia (HGD) and esophageal adenocarcinoma (EA) but at the expense of reduced sensitivity for metaplasia and dysplasia; absent staining in Sq, non-dysplastic Barrett's esophagus (NDBE), and low-grade dysplasia (LGD).
